# Influence of Ag nanoparticles anchored on protonated g-C_3_N_4_–Bi_2_MoO_6_ nanocomposites for effective antibiotic and organic pollutant degradation

**DOI:** 10.1039/d1ra02800f

**Published:** 2021-07-22

**Authors:** Muniyandi Govinda raj, Elayaperumal Vijayakumar, Bernaurdshaw Neppolian, Sandeep Kumar Lakhera, Aruljothy John Bosco

**Affiliations:** Department of Chemistry, SRM Institute of Science and Technology Kattankulathur 603203 Chennai Tamilnadu India johnbosa@srmist.edu.in sambosco@gmail.com +91-9840757430 +91-8610498493; Energy and Environmental Remediation Lab, SRM Research Institute, SRM Insitute of Science and Technology Kattankulathur 603 203 Chennai Tamilnadu India; Department of Physics and Nanotechnology, SRM Institute of Science and Technology Kattankulathur 603203 Chennai Tamilnadu India

## Abstract

The development of noble metal-anchored semiconductors for photocatalytic processes is now garnering interest for potential application to toxic pollutants as well as antibiotic degradation. Herein, we report novel Ag@p-g-C_3_N_4_–Bi_2_MoO_6_ nanocomposites synthesized by facile hydrothermal and calcination methods with a size of about 50 nm, exhibiting superior photocatalytic activity for charge separation. The resulting nanocomposites were evaluated by various physiochemical techniques such as X-ray diffraction, X-ray photoelectron spectroscopy, Fourier-transform infrared spectroscopy, scanning electron microscopy, and high-resolution transmission electron microscopy. The charge transfer photogenerated carriers were confirmed by photoluminescence spectra and electrochemical impedance spectroscopy. The anchoring of Ag nanoparticles over p-g-C_3_N_4_/Bi_2_MoO_6_ decreased the band gap energy from 2.67 to 2.48 eV, to exhibit an abnormal increase in absorption of light towards the visible light region. The degradation performance of the nanocomposites in terms of antibiotic ciprofloxacin and rhodamine B degradation efficiency was measured 85 and 99.7% respectively. The superoxide radical anion ˙O_2_^−^ played a significant role throughout the entire degradation process. Focusing on the probable mechanism based on the desirable results, the present work follows the heterostructure mechanism. Moreover, this work features the feasible applications of Ag@p-g-C_3_N_4_–Bi_2_MoO_6_ as a modified photocatalyst in the treatment of both domestic and industrial waste water.

## Abstract 2

The development of noble metal-anchored semiconductors for photocatalytic processes is now garnering interest for potential application to toxic pollutants as well as antibiotic degradation.

## Graphical abstract

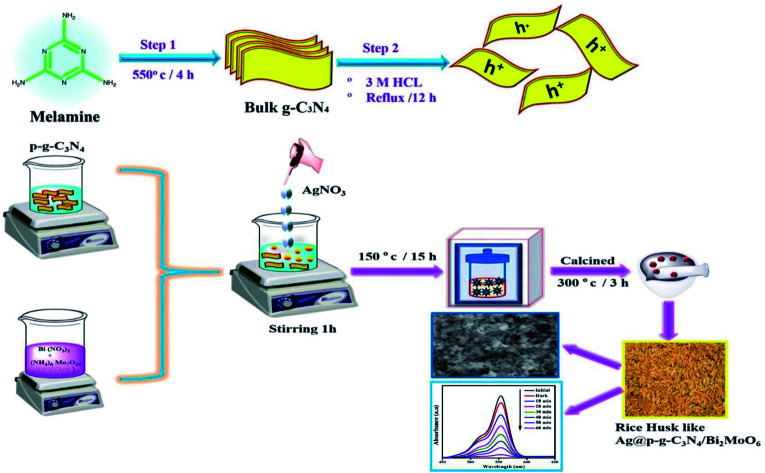

## 1. Introduction

Currently, there is a great demand for natural resources worldwide mainly due to environmental degradation and pollution.^1^ To resolve this issue, the provision of alternative renewable energy sources is required. Solar energy harvesting has received extensive attention in the fields of photocatalysis and energy storage applications.^2,3^ Due to the rapid development of industrial and human activities most of the carcinogenic dyes from tannery industries get passed directly into water, thereby resulting in environmental pollution.^4–6^ Among them, ciprofloxacin (CIP), C_17_H_18_N_3_O_3_, belongs to the second-generation fluoroquinolone antibiotic family mainly composed of quinolone structure and a piperazine moiety.^7^ CIP is extensively used in various fields, such as medicine, livestock and agriculture. Due to the metabolic activities, CIP has a negative impact on human health and the environment. To overcome these issues, antibiotics are being replaced by environmentally friendly and renewable technology. Photocatalysis offers an alternative solution for the complete degradation of antibiotics by boosting reaction conditions.^8–14^

Most of the traditional methods used for wastewater treatment have recently suffered due to them receiving less attention in environmental remediation, particularly anaerobic oxidation layer partition and adsorption.^15,16^ To overcome this issue, the use of a noble metal-based semiconductor photocatalyst has been found to be the most efficient and viable technique to degrade macromolecular contaminants into less harmful and non-toxic compounds. In general, an enormous number of semiconductor metal oxide and sulfide photocatalysts, including TiO_2_, ZnO, WO_3_, CdS, In_2_S_3_, and Ag_3_PO_4_, have been bench-marked for photocatalytic activity through the efficient degradation of organic pollutants.^17,18^

It is well known that polymeric graphitic carbon nitride (g-C_3_N_4_) with a layered 2D structure has a broad range of applications in materials chemistry due to its flexible physical and chemical properties and tunable electronic structure, showing outstanding potential for visible-light active photocatalysis.^19,20^ Besides this, g-C_3_N_4_ contains abundant functional groups such as carbon (C) and nitrogen (N), which augment the material properties to boost its broad specific area housing active absorption sites for organic pollutants.^21,22^ Meanwhile, there have been few attempts on investigating noble metals anchored on heterojunction semiconductors to reduce the band gap energy. Indeed, g-C_3_N_4_ has certain limitations in realistic photocatalysis applications, such as inadequate real surface area, less separation of photoexcited electron–hole pairs, limited electron transfer, poor quantum yield, and low light-harvesting ability. Promoting the efficiency of g-C_3_N_4_ by doping with transition metals (*e.g.*, Cu, Co, and Ag) and non-metal components (*e.g.* P, S, I, O, and B) not only improves the density of both donor and acceptor, but also facilitates the transport of electrons and increases the visible-light absorption at an appropriate wavelength.^23–26^ In recent reports the aurivillius metal oxide group is generally represented by the formula Bi_2_X_*n*−1_Y_*n*_O_3*n*+3_ (X = Ca, Sr, Ba, Pb, Na and Y = Ti, Nb, Ta, Mo, W) and is considered to be the most popular and active photocatalyst owing to its layered structures and outstanding photoelectrical properties. Bismuth molybdate (Bi_2_MoO_6_) consists of interlaced [Bi_2_O_2_]^2+^ and perovskite group (MoO_4_^2−^) layers.^27^ The foremost virtues of pure Bi_2_MoO_6_ are those such as lower band gap energy nearly equal to 2.4–2.8 eV, nontoxicity and low cost, and it showed an improved light response compared with wide-band-gap window photocatalysts such as TiO_2_ and ZnO. Moreover, pristine Bi_2_MoO_6_ suffers from a wide range of problems in large-scale practical applications, like poor separation of charge transfer, low quantum efficiency and scarce energetic sites.^28–30^ To resolve these disadvantages, numerous endeavors have been adopted involving modifying doping techniques.^31^

Considering the above key points, we are interested in synthesizing composites of Ag nanoparticles anchored on p-g-C_3_N_4_/Bi_2_MoO_6_ surface *via* a facile and straightforward hydrothermal method. As is evident, Ag nanoparticles affixed on the surface layer of p-g-C_3_N_4_/Bi_2_MoO_6_ can further improve the photoinduced charge carriers mainly due to surface plasmon resonance.^32^ Furthermore in comparison with bare g-C_3_N_4_/Bi_2_MoO_6_, decorating Ag nanoparticles on p-g-C_3_N_4_/Bi_2_MoO_6_ could enhance the photocatalytic degradation of various contaminants such as CIP and rhodamine B (RhB) under visible light irradiation, measured at around 85% and 99.7%, respectively, within 60 min.

## 2. Experimental

### 2.1 Materials

All reagents used in the present work were of analytical grade and used without further purification. Ultrapure water was used in all the experiments. Melamine (99%), bismuth(iii) nitrate pentahydrate, ammonium molybdate tetrahydrate, CIP, and RhB were purchased from Sigma-Aldrich, India. HCl (37 wt%) and ethanol used as solvents for synthesis were obtained from Avra Chemicals, India.

### 2.2 Synthesis of g-C_3_N_4_

For pristine g-C_3_N_4_, a modified procedure was adopted from a previous publication.^33^ Firstly, melamine was ground into fine powder with a mortar and pestle. The fine powder was transferred into an alumina boat crucible and kept for calcination at 550 °C at a ramp rate of 5 °C min^−1^ for 4 h. After the reaction was cooled to ambient temperature, the final yellow product was crushed and named as g-C_3_N_4_.

### 2.3 Synthesis of protonated g-C_3_N_4_ or p-g-C_3_N_4_

Typically, 1 g of bulk g-C_3_N_4_ was dissolved in 25 mL of 3 M HCl and stirred for 12 h at room temperature. After the completion of the reaction, the product suspension was washed with deionized water until it was neutral. The mixture was placed in a hot-air oven for drying at 80 °C for 12 h. Finally the dried powder was obtained and labelled as p-g-C_3_N_4_.^33^

### 2.4 Synthesis of Bi_2_MoO_6_

Bi_2_MoO_6_ was synthesized by a facile hydrothermal method with slight modification.^34^ About 0.485 g of Bi(NO_3_)_3_·5H_2_O and 0.088 g of Na_2_MoO_4_·2H_2_O were dissolved in 40 mL MilliQ-pore water under vigorous stirring for 5 h, followed by sonication for 15 min. The mixture was then transferred into a Teflon-lined stainless steel autoclave with a filling volume of 50 mL and heated up to 150 °C for 15 h. After being dried at room temperature the obtained product was washed with deionized water and absolute ethanol about three times and dried in a hot-air oven for 6 h at 80 °C. It was further annealed in a muffle furnace at 300 °C for 3 h (temperature ramp rate: 5 °C min^−1^).

### 2.5 Synthesis of p-g-C_3_N_4_/Bi_2_MoO_6_ and Ag@p-g-C_3_N_4_/Bi_2_MoO_6_

To fabricate the Ag-loaded nanocomposites, 100 mg of p-g-C_3_N_4_ was dissolved in 40 mL of deionized water and stirred for 30 min followed by the dropwise addition of 1 : 2 stoichiometric ratio of 0.485 g of Bi(NO_3_)_3_·5H_2_O and 0.088 g of Na_2_MoO_4_·2H_2_O. The solution was kept under vigorous magnetic stirring for 30 min. After that, for loading Ag into the bulk solution, prior as-weighed AgNO_3_ (0.0424 g) was added to above the mixture. Then, the solution was ultrasonically treated for 15 min under vigorous magnetic stirring for 1 h. It was then transferred into a Teflon-lined stainless steel autoclave of 50 mL capacity and heated up to 150 °C for 15 h. The obtained final product was centrifuged and washed with distilled water and absolute ethanol to remove the impurities and dried at 80 °C for 6 h. After drying, the samples were further annealed in a muffle furnace at 300 °C for 3 h (temperature ramp rate: 5 °C min^−1^). The obtained powder sample was denoted as Ag@p-g-C_3_N_4_/Bi_2_MoO_6_. For comparison, the same synthetic procedure was followed for p-g-C_3_N_4_/Bi_2_MoO_6_ but without addition of AgNO_3_.

### 2.6 Materials characterization

The crystallinity of the samples was probed using the Cu K alpha line (lambda = 0.154 nm) of an X-ray diffractometer (XRD, Panalytical Xpert Pro). XRD data were taken from 2*θ* = 10 to 80° with a step size of 0.025°. The obtained diffraction patterns were compared to a reference pattern.

Morphological studies were conducted to identify the structure of Ag@p-g-C_3_N_4_/Bi_2_MoO_6_ by scanning electron microscopy (SEM). The microscopic images were obtained using a Bruker FE-SEM operating at 15 000 V with a built-in EDX setup. High-resolution transmission electron microscopic (HRTEM) imaging and EDS elemental mapping were carried out with a JEOL 2010F TEM with an accelerating voltage of 200 kV. To confirm the chemical composition and oxidation state of the materials, X-ray photoelectron spectroscopy (XPS) was performed with a Physical Electronics System India, using Al Kα radiation as the excitation source. The obtained resultant binding energy values from XPS were calibrated with carbon peak as background reference. Fourier transform infrared spectroscopy (FTIR) was conducted with a Perkin Elmer instrument (USA) in the range of 4000 to 400 cm^−1^. The optical band gap and charge separation efficiency of the synthesized catalyst were analyzed by UV-visible DRS with BaSO_4_ as the background (Evolution 220 PC spectrophotometer). Photoluminescence (PL) analysis was performed with a Fluorolog (Horiba Yvon) spectrophotometer. A lamp source was used in the photocatalytic performance investigation (Xe lamp, 300 W). The transient photocurrent response of all of the synthesized samples was investigated on an electrochemical workstation using a general three-electrode setup (Shanghai Chenhua CHI-660D).

### 2.7 Photocatalytic experiments

The photodegradation of RhB dye and CIP antibiotic as model pollutants was investigated using a 300 W Xe lamp, with a UV cut-off filter (*λ* > 400 nm). Typically, 50 mg of Ag@p-g-C_3_N_4_/Bi_2_MoO_6_ photocatalyst was dispersed in 50 mL of pollutant solution (concentration of 10 ppm) and placed in a Pyrex reactor. For 30 min, the solution was stirred under dark condition to reach the adsorption–desorption equilibrium state for the solution. At regular intervals of 10 min, an aliquot of 4 mL was extracted at a particular time after visible light irradiation and the concentration of CIP antibiotic and RhB dye was determined by measuring the absorbance corresponding to their respective wavelengths (*λ*_max_) of 278 and 554 nm that was analyzed by a UV-visible spectrophotometer (Specord-200 plus UV-visible spectrophotometer, Germany). The photocatalyst and the remaining pollutant were separated using centrifugation. Furthermore, for recycling experiments, the photocatalyst cycling ability was investigated by analyzing the removal of CIP after the sequential running of four cycles. To identify the mineralization rate in the pollutant, total organic carbon (TOC) was measured using an analyzer (Shimadzu TOC-L instrument, Japan). Additionally, a trapping experiment was used to determine the significant active species involved in the degradation of the antibiotic. Different scavengers (concentration of 5 mM), namely benzoquinone (BQ), ethylenediaminetetraacetic acid disodium (EDTA-2Na), and isopropyl alcohol (IPA), were added into the CIP solution as scavengers of superoxide anion radicals, holes, and hydroxyl radicals, respectively.

## 3. Results and discussion

### 3.1 XRD analysis

**Fig. 1:**
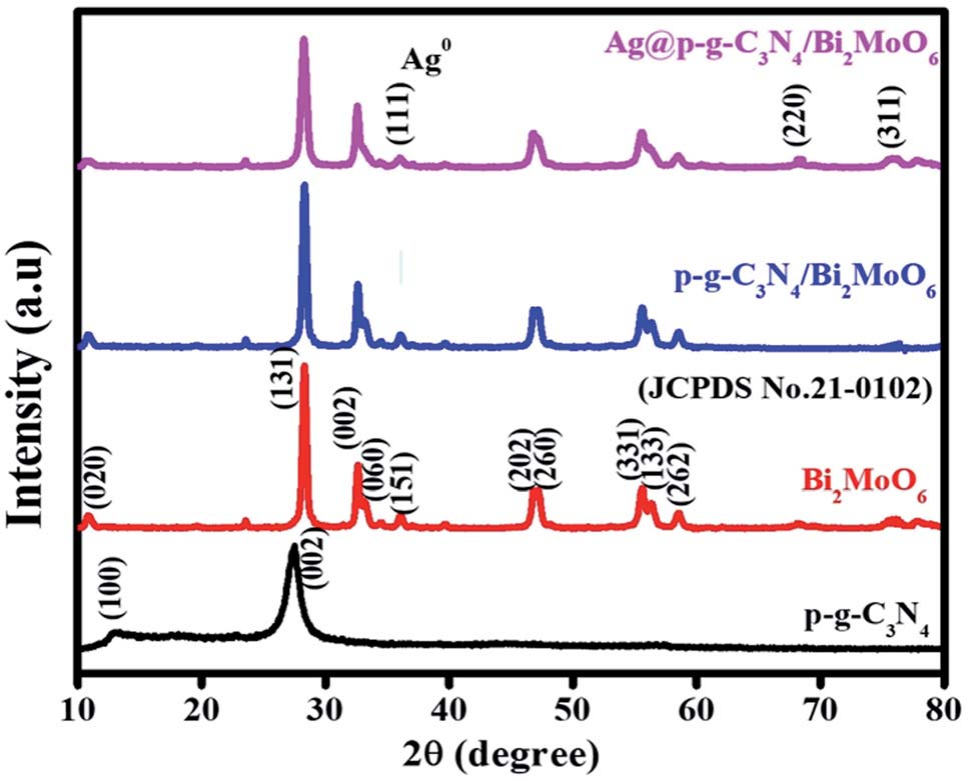
XRD patterns of the prepared p-g-C_3_N_4_, Bi_2_MoO_6_, p-g-C_3_N_4_/Bi_2_MoO_6_, and Ag@p-g-C_3_N_4_/Bi_2_MoO_6_ photocatalysts.

Powder XRD studies were used to investigate the crystalline structure of the as-prepared samples (p-g-C_3_N_4_, Bi_2_MoO_6_, p-g-C_3_N_4_/Bi_2_MoO_6_ and Ag@p-g-C_3_N_4_/Bi_2_MoO_6_). As seen in Fig. 1 the pristine p-g-C_3_N_4_ displays two distinct peaks at 2*θ* = 12.08° and 27.8°, which are due to the interplanar stacking corresponding to the tri-triazine groups [100] and [002] of the aromatic laminate plane, well matched to the standard JCPDS (87-1526) values.^35,36^ The XRD patterns are clearly defined to validate the orthorhombic crystalline structure of γ-BMO (JCPDS no. 21-0102). The diffraction peaks of Bi_2_MoO_6_ were observed in the pattern of p-g-C_3_N_4_/Bi_2_MoO_6_ composites, suggesting that heterojunctions were successfully established. The peaks of p-g-C_3_N_4_ of (100) and (002) phases were found to be invisible and overlapped with the (131) peak of Bi_2_MoO_6_. The characteristic diffraction peaks of Ag at 2*θ* = 32.7, 46.8, 55.7, and 67.9° suggest the (111), (200), (220), and (311) planes were observed for Ag-doped p-g-C_3_N_4_/Bi_2_MoO_6_ composites perfectly matching JCPDS no. 02-1067.^37^ Simultaneously, the rest of the major diffraction peaks of Bi_2_MoO_6_ show that Ag nanoparticles are decorated on the p-g-C_3_N_4_/Bi_2_MoO_6_ heterojunction.

### 3.2 XPS analysis

**Fig. 2:**
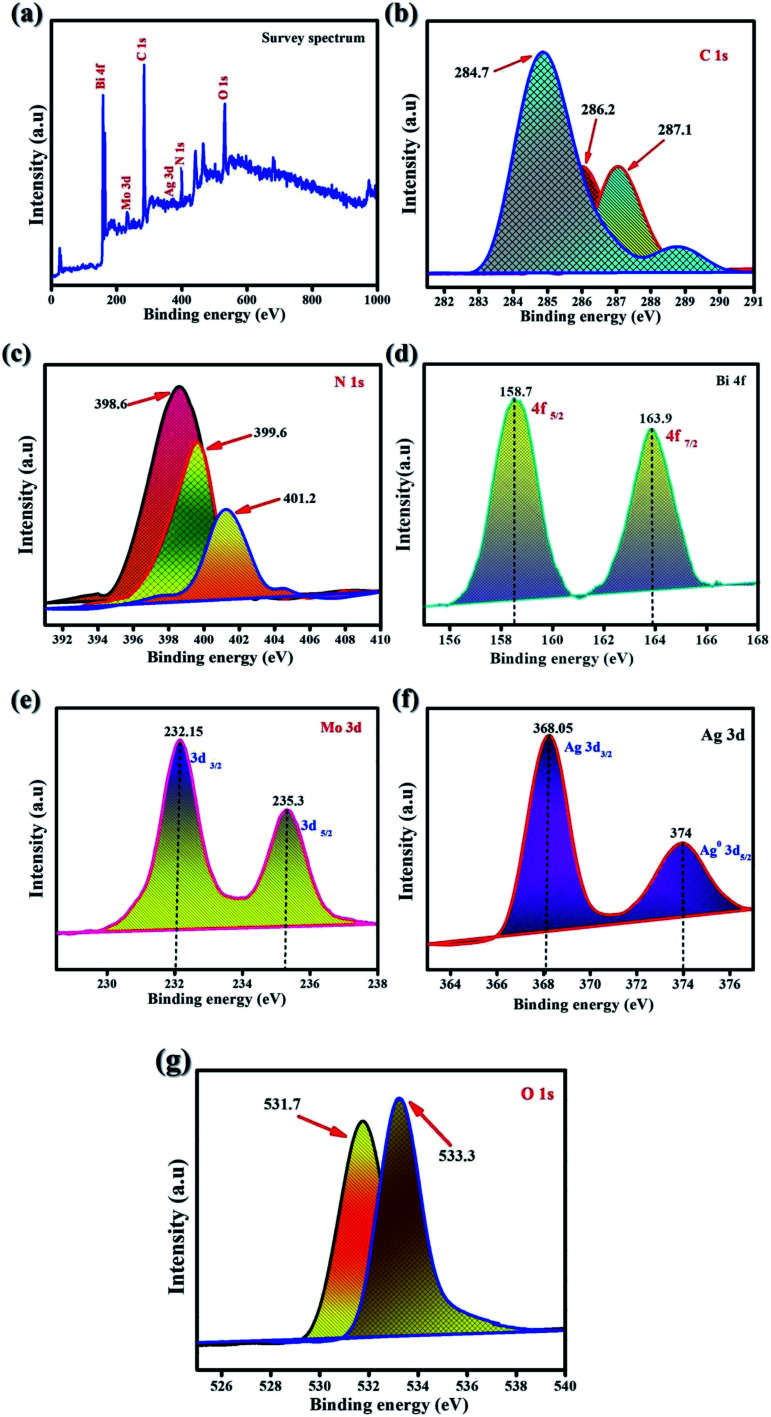
XPS spectra of the as-synthesized Ag@p-g-C_3_N_4_/Bi_2_MoO_6_. (a) Survey scan, (b) C 1s, (c) N 1s, (d) Bi 4f, (e) Mo 3d, (f) Ag 3d, and (g) O 1s.

XPS was performed to analyze the valency states and all chemical species of the synthesized samples. Fig. 2 presents the survey spectrum of all of the characteristic elements of carbon, nitrogen, bismuth, molybdenum, oxygen, and silver. Fig. 2a shows the XPS survey spectrum of as-synthesized samples of Ag@p-g-C_3_N_4_/Bi_2_MoO_6_. Fig. 2b shows the two distinct XPS peaks of C 1s, which could be attributed to amorphous carbon of graphite C–C bonds appearing at 284.7 and 287.1 eV. For p-g-C_3_N_4_ composite, the binding energies at 398.6 eV, 399.9 eV and 401.2 eV are observed for the N 1s spectrum as shown in Fig. 2c and fine characteristic peaks are attributed to C–N–C coordination. Bi 4f is detected *via* the signal as shown in Fig. 2d at consistent binding energies of 158.7 eV (Bi 4f_5/2_) and 163.9 eV (Bi 4f_7/2_), attributed to Bi metallic compound. The characteristic spin-orbital splitting of photoelectrons for Mo^6+^ oxidation state in the 3d orbital, particularly in d_5/2_ and d_3/2_, corresponds to the binding energy peaks in the range of 232.1 and 235.3 eV corresponding to individual Mo atoms that co-exist in different chemical states as shown in Fig. 2e The standard peaks of Ag 3d can be found in the elemental silver metal state as shown in Fig. 2f, the peaks at 367.8 and 374 eV corresponding to the Ag 3d_3/2_ and Ag 3d_5/2_ orbital regions, respectively. In comparison, the influential O 1s peaks can be seen in Fig. 2g at 531.7 and 533.3 eV, which is compatible with the MoO_6_ functionalization of Ag@p-g-C_3_N_4_/Bi_2_MoO_6_ nanocomposites.^38,39^

### 3.3 FTIR spectroscopy analysis

**Fig. 3:**
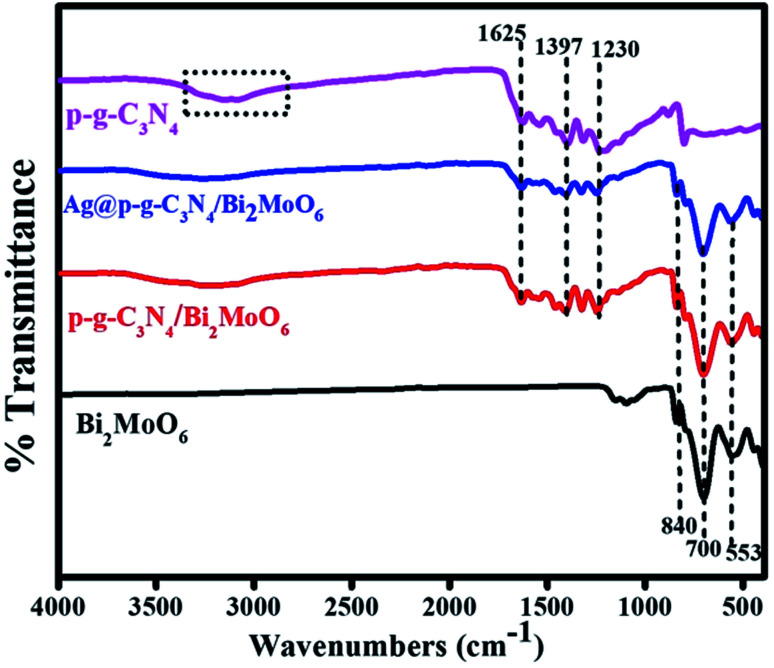
FTIR spectra of p-g-C_3_N_4_, Bi_2_MoO_6_, p-g-C_3_N_4_/Bi_2_MoO_6_, and Ag@p-g-C_3_N_4_/Bi_2_MoO_6_.

The presence of functional groups for as-prepared samples was further analyzed by FTIR. Fig. 3 shows the two pure sharp peaks at 800 cm^−1^ confirming the presence of bending mode of S-triazine in CN heterocycles. The peaks at 1230–1625 cm^−1^ were assigned to the stretching vibrations of C≡N. Moreover, the large bands at 1230, 1317 and 1397 cm^−1^ could be assigned to stretching vibration mode of C–N bond in the aromatic moiety. The peak at 1625 cm^−1^ corresponds to the stretching vibration mode of heptazine group. Additionally, the broad band at 3000–3500 cm^−1^ was ascribed to stretching mode of uncondensed amine N–H groups in g-C_3_N_4_. The pure Bi_2_MoO_6_ composite characteristic peaks at 553 and 700 cm^−1^ show the asymmetric and stretching vibration mode of the oxygen atoms in MoO_6_.^40^ The sharp bands at 700 and 840 cm^−1^ were attributed to the Mo–O stretching vibration mode in an octahedral structure.

### 3.4 SEM analysis

**Fig. 4:**
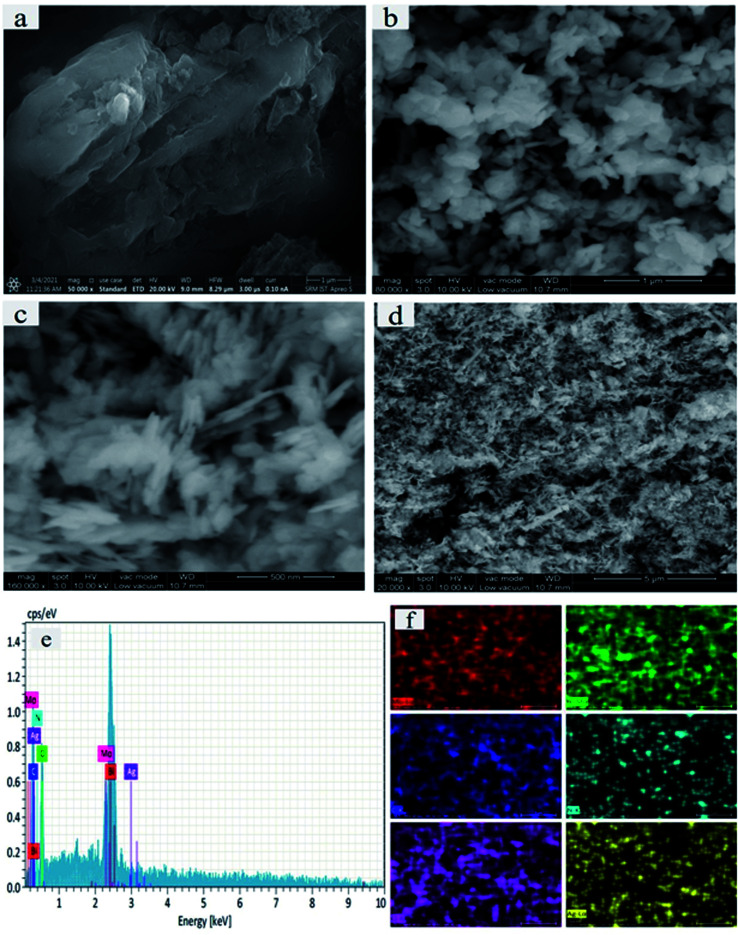
(a–d) SEM images of as-synthesized samples: (a) p-g-C_3_N_4_, (b) Bi_2_MoO_6_, (c) p-g-ssC_3_N_4_/Bi_2_MoO_6_ and (d) Ag@p-g-C_3_N_4_/Bi_2_MoO_6_. (e) Elemental composition of Ag@p-g-C_3_N_4_/Bi_2_MoO_6_. (f) Elemental maps of Ag@p-g-C_3_N_4_/Bi_2_MoO_6_.

The surface morphologies and microstructural elemental composition of the as-prepared samples were further examined by SEM. Fig. 4a–d displays a large surface area of p-g-C_3_N_4_ wrinkled 2d morphology doping with p-g-C_3_N_4_/Bi_2_MoO_6_. Fig. 4a depicts the ultrathin uniform bulk layer of pristine p-g-C_3_N_4_. Notably, the pure Bi_2_MoO_6_ micro-petals with an irregular plate-like microstructure are shown in Fig. 4b. Moreover, the microplate combination of both p-g-C_3_N_4_ and Bi_2_MoO_6_ nanocomposites is shown in Fig. 4c. Interestingly, Fig. 4d shows the final composite of Ag nanoparticles anchored on p-g-C_3_N_4_ and Bi_2_MoO_6_ thereby confirming that some amount of Ag nanoparticles were loaded into the heterostructure, sufficiently enhancing the photocatalytic activity. Interestingly the final catalyst Ag@p-g-C_3_N_4_/Bi_2_MoO_6_ shows a rice husk-like morphology. Fig. 4e shows the EDS spectrum of the final compositions of the prepared nanocomposites. This also indicates that all of the elements C, N, Mo, Bi and Ag are present in the final composition, and the heterostructure of Ag@p-g-C_3_N_4_/Bi_2_MoO_6_ had been effectively fabricated. Fig. 4f shows the elemental mapping of Ag@p-g-C_3_N_4_/Bi_2_MoO_6_, with all the elements being present in the final composition.

### 3.5 TEM analysis

**Fig. 5:**
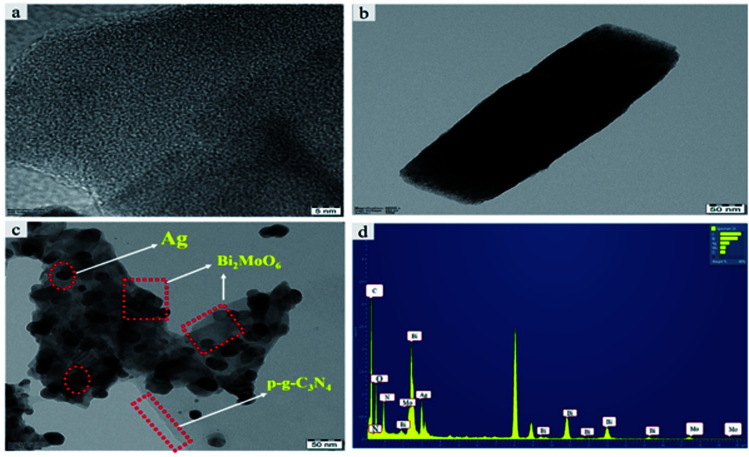
Typical TEM images of (a) pristine p-g-C_3_N_4_, (b) Bi_2_MoO_6_, and (c) Ag@p-g-C_3_N_4_/Bi_2_MoO_6_. (d) EDX spectrum of Ag@p-gC_3_N_4_/Bi_2_MoO_6_.

**Fig. 6:**
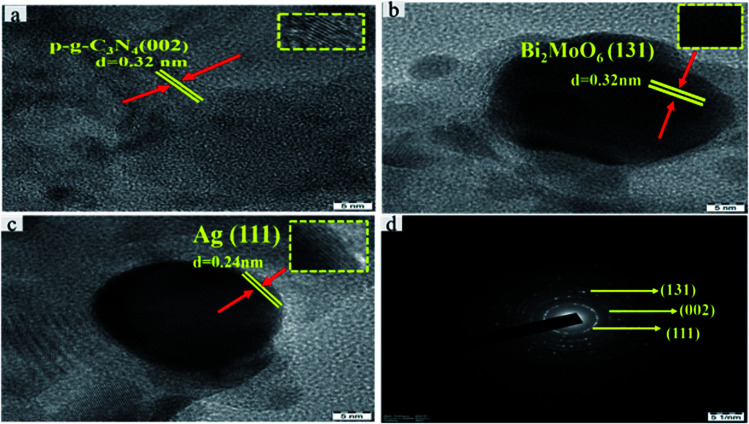
HRTEM images of (a) p-g-C_3_N_4_, (b) Bi_2_MoO_6_, and (c) Ag. (d) Selected area electron diffraction pattern for Ag@p-g-C_3_N_4_/Bi_2_MoO_6_.

To further investigate the final heterostructured surface morphology, TEM was carried out for synthesized p-g-C_3_N_4_, Bi_2_MoO_6_ and Ag@p-g-C_3_N_4_/Bi_2_MoO_6_ nanocomposites as depicted in Fig. 5. As illustrated in Fig. 5a the TEM image of p-g-C_3_N_4_ shows a layered nanosheet, with an average thickness of 6–10 nm. Fig. 5b reveals a lower magnification image of Bi_2_MoO_6_ nanopetal. It is worth noting that Fig. 5c shows a close-up view of dark spot highlighted in red of Ag enwrapped with nanoplates of Bi_2_MoO_6_ and p-g-C_3_N_4_ nanosheet, which strongly confirms the formation of Ag@p-gC_3_N_4_/Bi_2_MoO_6_. Fig. 5d confirms the EDX spectrum for Ag@p-g-C_3_N_4_/Bi_2_MoO_6_. This clearly shows that all the chemical components of Ag@p-g-C_3_N_4_/Bi_2_MoO_6_ derived rice husk composites contain Ag, Bi, C, N, O, and Mo elements. Furthermore, HRTEM images of pure p-g-C_3_N_4_, Bi_2_MoO_6_, and Ag@p-g-C_3_N_4_/Bi_2_MoO_6_ are displayed in Fig. 6. It is clear from Fig. 6a that the boxed area shows the interplanar spacing lattice fringes of about 0.32 nm corresponding to the (002) facets of p-g-C_3_N_4_ and is in good agreement with the XRD pattern. Similarly Fig. 6b displays the selected part of Bi_2_MoO_6_ highlighted in red color. It also shows the interplanar spacing lattice fringes of about 0.32 nm corresponding to the (131) plane of Bi_2_MoO_6_. Likewise, Fig. 6c shows the interplanar spacing lattice fringes of about 0.240 nm corresponding to the (111) plane of Ag. The selected area electron diffraction pattern in Fig. 6d reveals selected discrete spots indexed to (131), (002) and (111) of the planes corresponding to Ag@p-g-C_3_N_4_/Bi_2_MoO_6_.

### 3.6 UV-visible diffuse reflectance spectroscopy analysis

**Fig. 7:**
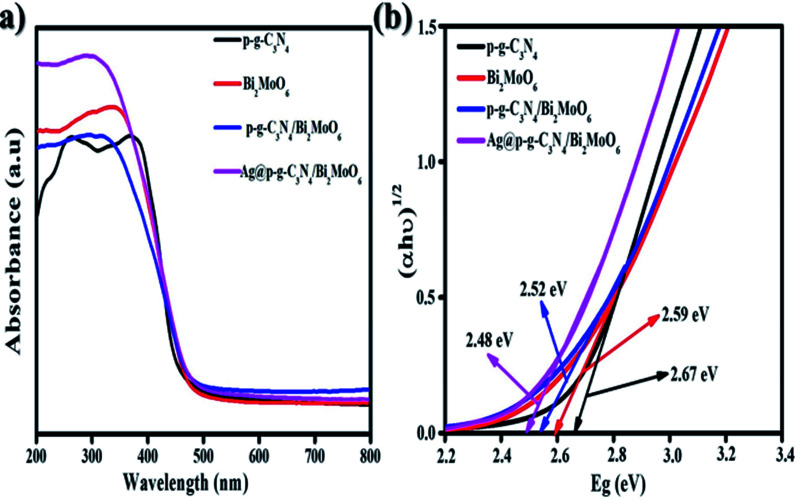
(a) UV diffuse reflectance spectra and (b) graph of (*αhν*)^1/2^*versus* energy (eV) of the as-synthesized Ag@p-gC_3_N_4_/Bi_2_MoO_6_ photocatalyst.

To examine the absorption ranges and optimum energy band gaps of the synthesized nanocomposites, the optical absorption was examined from 200 to 800 nm, as shown in Fig. 7a. The p-g-C_3_N_4_ exhibited a moderate absorption edge around 435 nm while the bare Bi_2_MoO_6_ showed a steep absorption edge that is found to be 470 nm. When p-g-C_3_N_4_ was intercalated with Bi_2_MoO_6_, outstanding peaks were observed in the region of 550 nm and the absorption edges became steeper due to p-g-C_3_N_4_ intercalation properties. After loading Ag nanoparticles onto the surface of p-g-C_3_N_4_/Bi_2_MoO_6_, the absorption edge exhibited higher absorption intensities than that for bare p-g-C_3_N_4_, Bi_2_MoO_6_, and p-g-C_3_N_4_/Bi_2_MoO_6_. From the DRS studies it can be clearly understood that incorporating Ag onto p-g-C_3_N_4_/Bi_2_MoO_6_ extends the light absorption into the visible region thus contributing to the great potential for photocatalytic degradation. The energy gaps were determined using Tauc plots of *αhν* = *A*(*hν* − *E*_g_)^1/2^*versus* photon energy as shown in Fig. 7b, where *α* = absorption coefficient, *h* = Planck's constant, *ν* = light frequency and *E*_g_ = band gap energy. With the excellent agreement of the curve the obtained band gap energy (*E*_g_) value was evaluated at 2.48 eV. The final catalyst was found to possess a direct band gap based on a literature survey.^41^

### 3.7 Photoluminescence spectra analysis

**Fig. 8:**
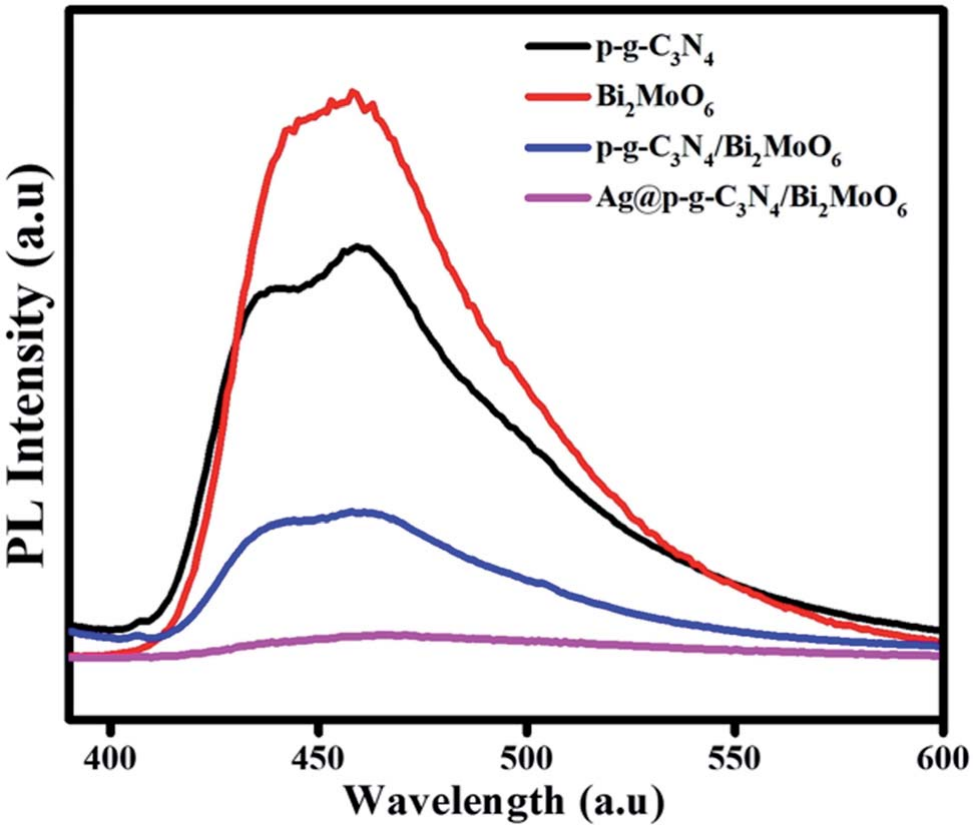
PL spectra of p-g-C_3_N_4_, Bi_2_MoO_6_, p-g-C_3_N_4_/Bi_2_MoO_6_, and Ag@p-g-C_3_N_4_/Bi_2_MoO_6_.

The PL emission spectra of pure p-g-C_3_N_4_, Bi_2_MoO_6_ and combinations of p-g-C_3_N_4_/Bi_2_MoO_6_ and Ag@p-g-C_3_N_4_/Bi_2_MoO_6_ catalysts were recorded under an excitation irradiation wavelength of 400 nm as shown in Fig. 8. PL is an important technique to investigate the interfacial charge transfer and isolation of photogenerated electrons and holes in photocatalysts. A lower PL intensity of Ag@p-g-C_3_N_4_/Bi_2_MoO_6_ than pure p-g-C_3_N_4_ and Bi_2_MoO_6_ can be observed from the PL spectra. This may be due to conduction band levels of bare p-g-C_3_N_4_ and Ag@p-g-C_3_N_4_ that stand at equal positions, quickly suppressing the rate of charge carrier recombination. Besides, it is interesting to note when Ag nanoparticles were decorated on the surface of p-g-C_3_N_4_/Bi_2_MoO_6_, this reduced the recombination rate of photogenerated electron–hole pairs and exhibited the lowest emission intensity, which greatly enhanced the photocatalytic activity due to the surface plasmon resonance effect of Ag.

### 3.8 BET analysis

**Fig. 9:**
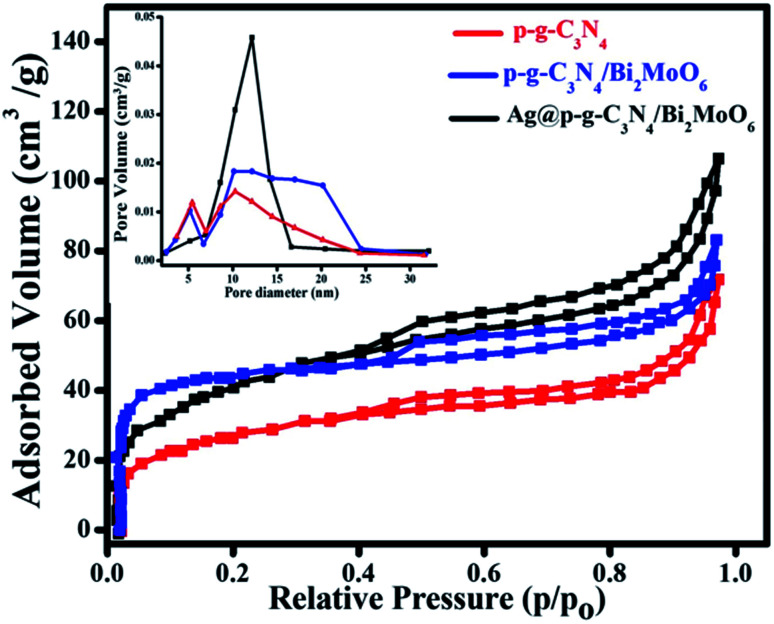
N_2_ adsorption–desorption isotherms and the corresponding (inset) pore-size distribution curves for as-prepared nanocomposites.

As seen in Fig. 9, the Brunauer–Emmett–Teller (BET) technique was used to quantify the precise surface area and pore size distributions of the prepared nanocomposites under N_2_ adsorption–desorption equilibrium conditions.^42^ The surface area of p-g-C_3_N_4_, p-g-C_3_N_4_/Bi_2_MoO_6_, and Ag@p-g-C_3_N_4_/Bi_2_MoO_6_ nanocomposites was found to be 43.5, 64.2, and 98.4 cm^3^ g^−1^, according to the BET analysis results. The broad surface area of the Ag@p-g-C_3_N_4_/Bi_2_MoO_6_ photocatalyst shows that the recombination rate of photogenerated charge carriers has been reduced. Furthermore, the increased pore volume and diameter, as well as better isolation and migration of photogenerated charges, contributes to increased photocatalytic activity. As a consequence of the above results, the main Ag@p-g-C_3_N_4_/Bi_2_MoO_6_ composite is useful for improved adsorption and also provides a greater number of reactive sites for photocatalytic processes, with a major effect on photocatalytic efficiency development.^43,44^ Importantly, in addition to the obvious increase in surface area, an increase in pore volume (inset of Fig. 9) was found, rising from 0.15 cm^3^ g^−1^ for as-prepared p-g-C_3_N_4_ to 0.46 cm^3^ g^−1^ for Ag@p-g-C_3_N_4_/Bi_2_MoO_6_, facilitating charge separation in the entire composite structure. Additionally, the addition of effective Ag and Bi_2_MoO_6_ components increased the pore diameter from 10.6 nm for as-prepared p-g-C_3_N_4_ to 15.2 nm for Ag@p-g-C_3_N_4_/Bi_2_MoO_6_.

### 3.9 Photoelectrochemical analysis

**Fig. 10:**
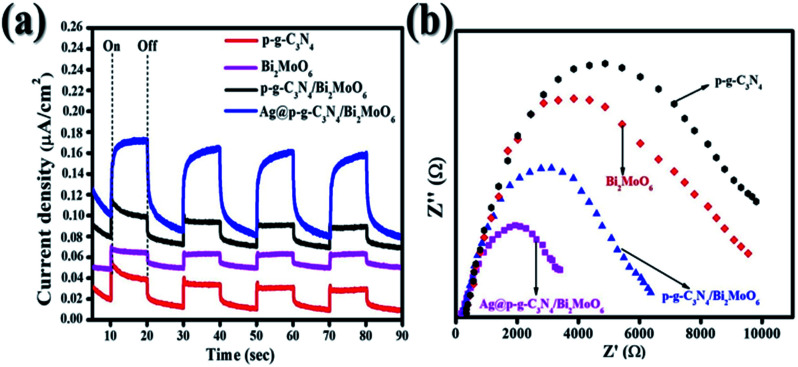
(a) Transient photocurrent response of all synthesized samples and (b) EIS curves of all synthesized samples.

Photocurrent measurements and electrochemical impedance spectroscopy (EIS) are valuable methods for characterizing the separation and migration potential of photoexcited charges. As shown in Fig. 10a, the transient photocurrent measurements of Ag@p-g-C_3_N_4_/Bi_2_MoO_6_ indicate a high photocurrent density compared with pure samples, implying a more effective separation of the photoexcited charges (electron–hole pairs) and limitation of their recombination. Several repeat on–off cycles display a similar photocurrent reaction. Significantly, it shows that the samples have high photostability. EIS electrochemical measurements of the as-synthesized samples were also used to analyze the charge-transfer resistance between the heterostructures. Fig. 10b depicts the smaller semicircle radius of prepared nanocomposites than the pure p-g-C_3_N_4_ and Bi_2_MoO_6_. This indicates the higher efficacy in separation and charge transfer of photogenerated electron–hole pairs.

### 3.10 Photocatalytic performance

**Fig. 11:**
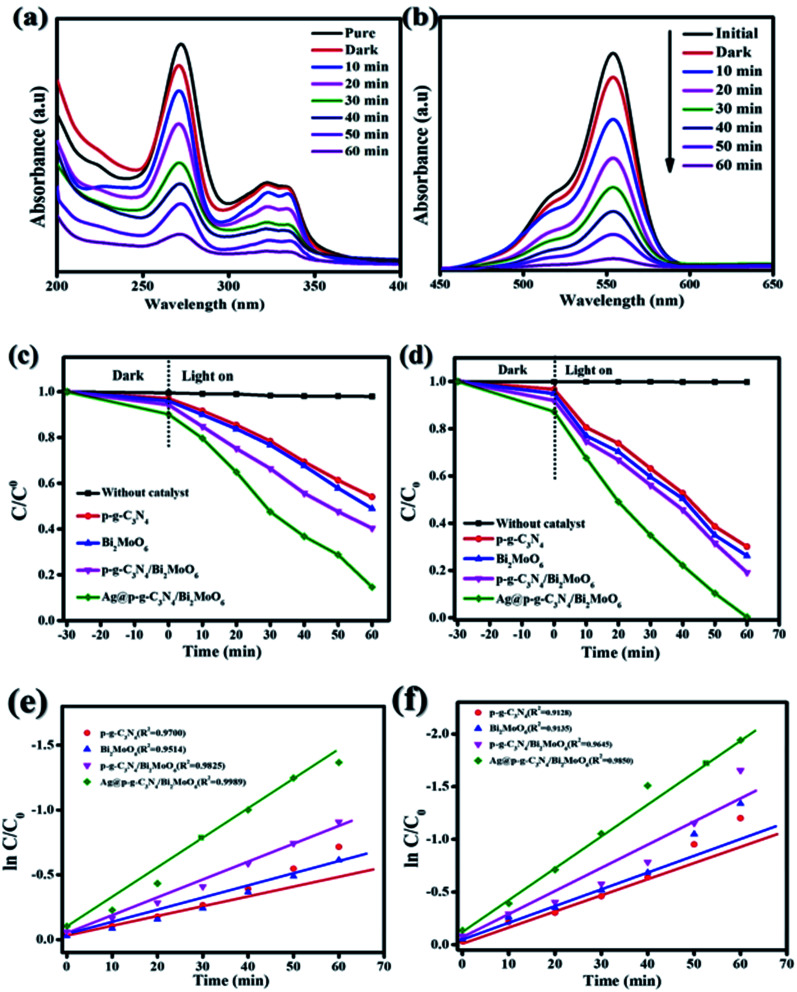
(a and b) Degradation of CIP and RhB over Ag@p-g-C_3_N_4_/Bi_2_MoO_6_ nanocomposite. (c and d) Photocatalytic CIP and RhB degradation for all synthesized nanocomposites. (e and f) Corresponding kinetic plots (*k*_obs_) of the as-synthesized nanocomposites.

The photocatalytic activity of Ag@g-C_3_N_4_/Bi_2_MoO_6_ was investigated in the degradation of CIP and RhB under visible light irradiation. The results are shown in Fig. 11a and b. The results of the degradation of CIP and RhB using Ag@g-C_3_N_4_/Bi_2_MoO_6_ show excellent photocatalytic performance, with values of 85% and 99.7%, which are much higher than when using pure p-g-C_3_N_4_, Bi_2_MoO_6_ and p-g-C_3_N_4_/Bi_2_MoO_6_ combination. When pure g-C_3_N_4_ is used under visible light, it shows poor photocatalytic activity and this may be due to the inhibitory effect on the surface area of graphene sheets, while g-C_3_N_4_ loaded on the metallic surface of Bi_2_MoO_6_ would allow effective separation of the electron–hole pairs. In addition, Ag nanoparticles enwrapped on p-g-C_3_N_4_/Bi_2_MoO_6_ would further increase the photocatalytic degradation effect due to the surface plasmon resonance interaction between Ag and p-g-C_3_N_4_. All synthesized catalyst activities are shown in Fig. 11c and d. To understand the decomposition rate, the kinetic behavior of CIP and RhB degradation is represented as a plot of *C*/*C*_0_*versus* time (min) in accordance with the pseudo first-order reaction kinetics based on the following equation:−ln(*C*/*C*_0_) =*Kt*where *C* is the concentration of pollutants at time *t*, *C*_0_ is the initial concentration of CIP and RhB, and *K* is the reaction rate constant as shown in Fig. 11e and f.

### 3.11 Radical trapping experiments

**Fig. 12:**
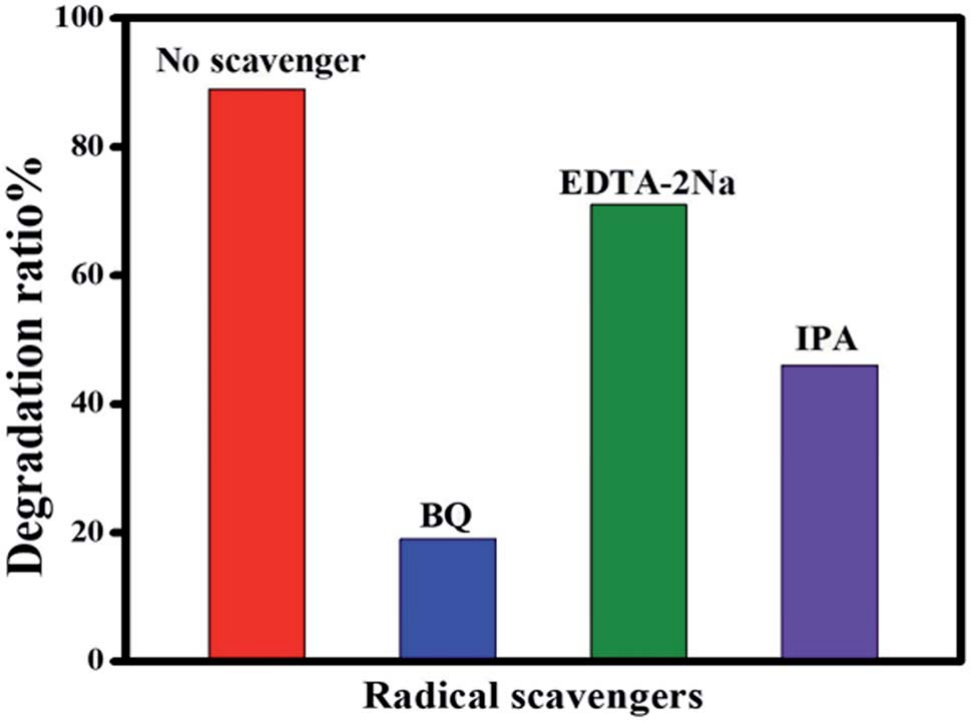
Effect of scavengers on the photocatalytic degradation of CIP over Ag@p-g-C_3_N_4_/Bi_2_MoO_6_ nanocomposite.

To identify the reactive species involved in the photodegradation of CIP, a series of radical trapping experiments were carried out using various radical scavengers. IPA was chosen as a quencher of hydroxyl radicals (˙OH), EDTA-2Na was selected as a quencher of holes (h^+^) and BQ was chosen as a quencher of the superoxide radical anion (˙O_2_^−^). These quenchers were added to the photocatalyst during the degradation process. As can be seen in Fig. 12, when BQ was added into the reaction mixture the degradation rate achieved was only 19%. This clearly shows that BQ has a strong effect on CIP degradation, which demonstrates that (˙O_2_^−^) plays an essential role in the degradation process. In contrast, the addition of IPA and EDTA-2Na has not much effect on CIP degradation, clearly indicating that (h^+^) and (˙OH) do not have much impact on the photocatalytic system.

### 3.12 Plausible degradation mechanism

**Fig. 13:**
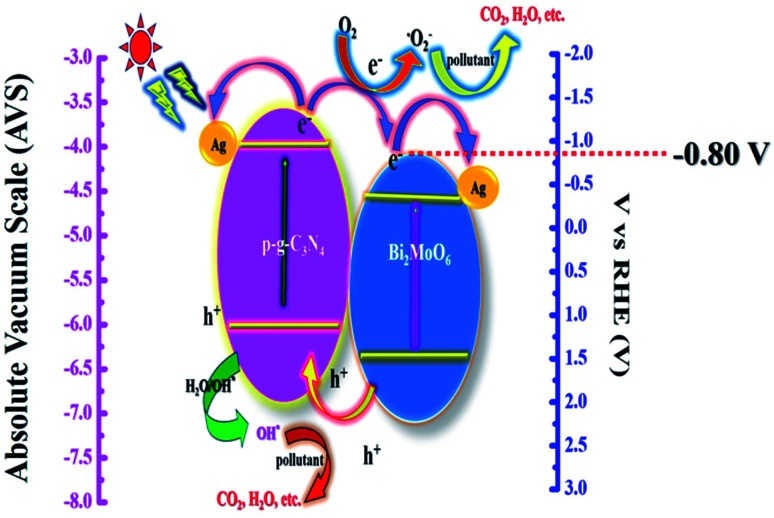
Schematic representation of the probable photocatalytic mechanism for Ag@p-g-C_3_N_4_/Bi_2_MoO_6_.

The suggested photocatalytic mechanism of the synthetic heterojunctions is depicted in Fig. 13. Upon visible light irradiation, both semiconductors p-g-C_3_N_4_ and Bi_2_MoO_6_ are excited to produce photogenerated electron–hole pairs. The diagram demonstrates clearly that ˙OH plays a less active role in the dye degradation process due to the band gap level of photogenerated h^+^ having an energy of 2.48 V in the valence band level showing a lower energy gap than ˙OH with an average energy of 2.70 V, suggesting that it is difficult for ˙OH to interact from H_2_O during photodegradation. It is worth noting that h^+^ has a strong oxidation ability which directly decomposes the CIP molecule. More particularly, e^−^ at the conduction band is much smaller (−0.80 V) than that of ˙O_2_^−^ which is 1.23 V, resulting in a simple reduction of dissolved O_2_ to ˙O_2_^−^ which degrades various organic matter.^45^ More particularly, the decorated Ag nanoparticles used in this work play an important role in serving as a solid-state electron mediator. The e^−^ developed on the conduction band of p-g-C_3_N_4_ heterojunctions is easily transferred to Ag particles and Bi_2_MoO_6_ microplates, effectively promoting electron transfer and suppressing electron–hole recombination efficiency.^33^ Furthermore, the formed h^+^ could migrate from Bi_2_MoO_6_ to g-C_3_N_4_ in the heterojunctions, reducing recombination efficiency significantly. As a result, the photocatalytic dye degradation efficiency of Ag@p-g-C_3_N_4_/Bi_2_MoO_6_ is higher than those of pure p-g-C_3_N_4_, Bi_2_MoO_6_, and p-g-C_3_N_4_/Bi_2_MoO_6_ composites.

### 3.13 TOC analysis

**Fig. 14:**
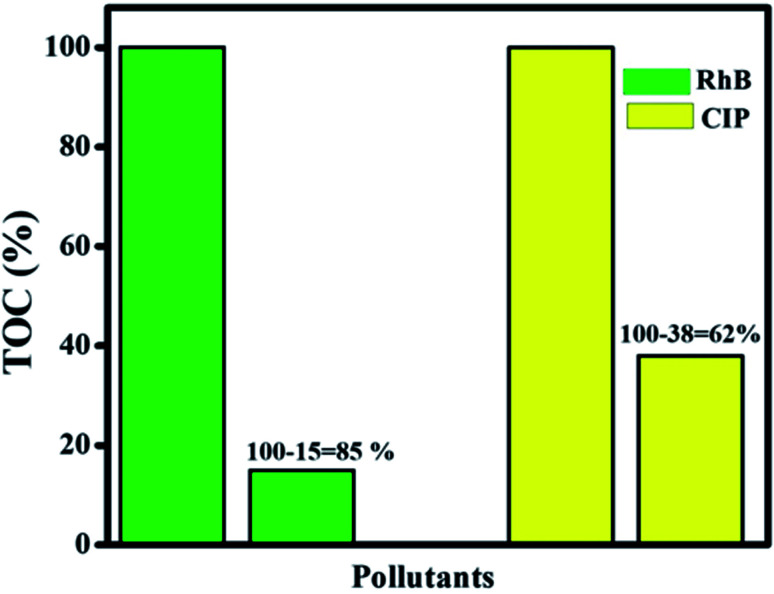
Mineralization efficiency for RhB and CIP of the final Ag@p-g-C_3_N_4_/Bi_2_MoO_6_ catalyst.

The TOC technique was employed to assay the mineralization of CIP and RhB using the optimum photocatalyst. Fig. 14 shows that mineralization efficiency values of 62% and 85% were achieved for CIP and RhB, respectively, while the photocatalytic efficiency values were 85% and 99.7% for CIP and RhB under 60 min of irradiation. This difference between mineralization and photocatalytic efficiency is due to the intermediates present during the photocatalytic process that can be mineralized in a longer reaction time.

### 3.14 Recyclability and stability

**Fig. 15:**
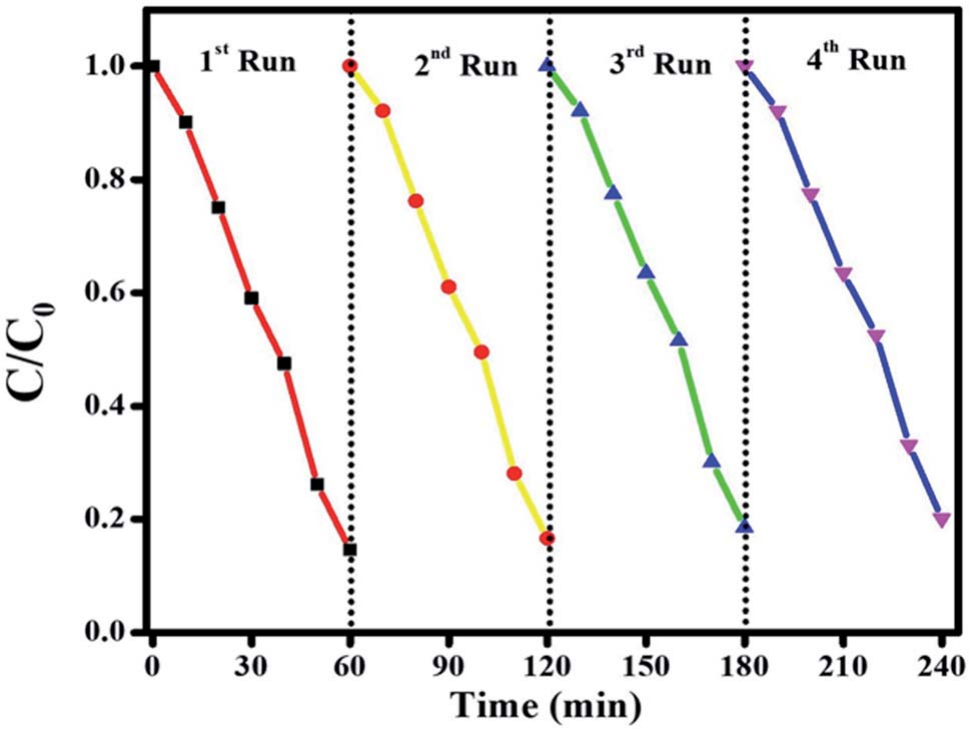
The stability test for CIP degradation activity for the final Ag@p-g-C_3_N_4_/Bi_2_MoO_6_ catalyst.

**Fig. 16:**
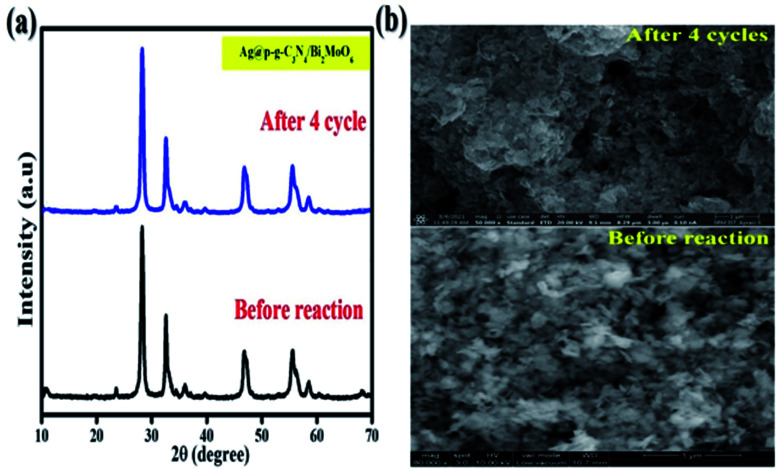
(a) XRD patterns and (b) SEM images of fresh and used Ag@p-g-C_3_N_4_/Bi_2_MoO_6_ catalyst.

To check the stability of the photocatalyst, the recyclability is a vital parameter to determine for the final nanocomposite. Fig. 15 shows the results of stability tests for CIP degradation activity for the final Ag@p-g-C_3_N_4_/Bi_2_MoO_6_ catalyst. After 4 consecutive runs, the degradation efficiency of the catalyst was found to be 92%, which indicates the high stability of the prepared photocatalyst. For the sake of comparison, the stability of the photocatalyst was evaluated by powder XRD and SEM after four successive repeat runs and the results are displayed in Fig. 16a and b. XRD patterns and SEM images of fresh and used samples reveal that the catalyst's crystalline nature and morphological features are not destroyed after several cycles.

## 4. Conclusion

To conclude, we developed a simple and environmentally friendly approach of using a ternary photocatalyst, Ag@p-g-C_3_N_4_/Bi_2_MoO_6_, for environmental contaminant degradation. The unique characterization techniques revealed that the prepared Ag@p-g-C_3_N_4_/Bi_2_MoO_6_ nanocomposite possessed high purity and crystallinity. It is also to be noted that the interfacial charge separation and efficiency are fully controlled due to the formation of interfacial bonds between metallic Ag and p-g-C_3_N_4_/Bi_2_MoO_6_. Moreover, the Ag@p-g-C_3_N_4_/Bi_2_MoO_6_ catalyst shows enough durability for the degradation of CIP. Ag nanoparticles were decorated on p-g-C_3_N_4_/Bi_2_MoO_6_ which leads to a higher surface area of the photocatalyst for the photodegradation of CIP and RhB under visible light irradiation for a duration of 1 h. After four cycles, the photocatalyst maintains its effectiveness and exhibits excellent structural stability. The pollutant undergoes a pseudo first-order reaction. The mechanistic pathway suggests that (˙O_2_^−^) plays a significant role in the photodegradation of the various pollutants. Thus, the present study demonstrates that capped Ag@p-gC_3_N_4_/Bi_2_MoO_6_ photocatalysts have a high potential for use in environmental protection.

## Conflicts of interest

There are no conflicts to declare.

## Supplementary Material
